# Hospital Saturday Fund

**Published:** 1886-11-06

**Authors:** 


### HOSPITAL SATURDAY FUND.

Mr. H. N. Hamilton-Hoare writes: "In consequence of the death of the late Mr. Samuel Morley, it devolves upon me, as treasurer of the movement, to request the favour of an intimation through your columns of the approaching close of the books for the present year, and to solicit the co-operation especially of those employers and foremen who have not yet organised a collection in aid of this fund at their various establishments. The friends of this movement regard it as a healthy sign that notwithstanding the long-continued depression of trade and industry in all its branches, the amount collected has continued to increase every year since the establishment of the fund. A most promising feature in this year's collection is the increased support accorded by employers of labour, who have in many instances supplemented by donations the amount collected by their employés; and I would suggest that, remembering the pressing requirements of the great majority of the hospitals and dispensaries of London, many not immediately connected with the industrial classes would be willing to add their contributions to this year's collections. There is no reason why the fund should be limited to amounts collected in the workshops; such a fund might fairly embrace the contributions of all not reached by the Sunday Fund—such, we believe, was the desire and intention of the founders of this movement. During the past twelve years 'Hospital Saturday' has contributed nearly £75,000 to the medical charities of London; during the present year up to this date, upwards of 2,000 receipts have been issued, and the amount in hand is considerably in excess of the amount in hand at the same date in any former year.

"Upon the governing body of the fund, there are representatives of most of the important warehouses and factories of London, and in addition to the original purpose of the Hospital Saturday collection, as in the case of most large movements, the strengthening of our organisation has led up to the adoption of other modes of usefulness to that class most interested in the success of our association; such as the establishment of the Seaside Convalescent Home for Working Men, at St. Margaret's Bay, Dover, where over 1,000 men have been admitted during the past three years, and such as the scheme for the supply of surgical appliances at the offices of our fund, were working satisfactorily. In making this appeal, permit me to add that the collectors in the various workshops—and all who hold our receipts—have the right of recommending those needy and deserving cases which come under their notice for medical or surgical assistance at any of the 130 participating institutions. Contributions in aid of the fund may be forwarded to me at this address, or to the secretary, 41, Fleet Street, and will be duly acknowledged. All amounts to hand before the end of November will be included in the thirteenth annual report."

41, Fleet Street, E.C.

